# Overexpression of the MRI Reporter Genes Ferritin and Transferrin Receptor Affect Iron Homeostasis and Produce Limited Contrast in Mesenchymal Stem Cells

**DOI:** 10.3390/ijms160715481

**Published:** 2015-07-08

**Authors:** Sofia M. Pereira, Diana Moss, Steve R. Williams, Patricia Murray, Arthur Taylor

**Affiliations:** 1 Institute of Translational Medicine, University of Liverpool, Liverpool L69 3BX, UK; E-Mails: sofiamp@liverpool.ac.uk (S.M.P.); dijim@liverpool.ac.uk (D.M.); p.a.murray@liverpool.ac.uk (P.M.); 2 Centre for Imaging Sciences, Oxford Road, University of Manchester, Manchester M13 9PT, UK; E-Mail: steve.williams@manchester.ac.uk

**Keywords:** reporter genes, magnetic resonance, biogenic nanoparticles, cell tracking, chick embryo, ferritin, transferrin receptor

## Abstract

Imaging technologies that allow the non-invasive monitoring of stem cells *in vivo* play a vital role in cell-based regenerative therapies. Recently, much interest has been generated in reporter genes that enable simultaneous monitoring of the anatomical location and viability of cells using magnetic resonance imaging (MRI). Here, we investigate the efficacy of ferritin heavy chain-1 (*Fth1*) and transferrin receptor-1 (*TfR1*) as reporters for tracking mesenchymal stem cells. The overexpression of *TfR1* was well tolerated by the cells but *Fth1* was found to affect the cell’s iron homeostasis, leading to phenotypic changes in the absence of iron supplementation and an upregulation in transcript and protein levels of the cell’s endogenous transferrin receptor. Neither the sole overexpression of *Fth1* nor *TfR1* resulted in significant increases in intracellular iron content, although significant differences were seen when the two reporter genes were used in combination, in the presence of high concentrations of iron. The supplementation of the culture medium with iron sources was a more efficient means to obtain contrast than the use of reporter genes, where high levels of intracellular iron were reflected in transverse (T_2_) relaxation. The feasibility of imaging iron-supplemented cells by MRI is shown using a 3R-compliant chick embryo model.

## 1. Introduction

The development of imaging techniques that allow the monitoring of a cell’s anatomical location *in vivo* has seen great progress in recent years. Scientists now have a variety of instrumentation that allows detection *in vivo* of cells labelled with fluorescent, bioluminescent, radioactive or magnetic probes [1,2,3]. Such techniques are of great importance in regenerative medicine and cancer research, as they enable one to image the dynamics of cell migration and engraftment. Magnetic resonance imaging (MRI) is an important tool for cellular imaging as it is translatable to the clinic. An example of its potential are the reports on tracking cells in human patients, where cells have been labelled *in vitro* with iron oxide based contrast agents, and then imaged following their administration [4]. Despite these encouraging results, iron oxide based labelling of cells can suffer from some drawbacks such as dilution of the contrast agent upon cell division, effectively leading to a loss of signal, or the presence of false positives. The latter happens when the administered cells die and the contrast agent is taken up by the host cells, in which case the signal no longer correlates with the cells of interest [3].

In order to overcome the weaknesses associated with iron oxide based cell labelling, efforts have been geared towards the development of reporter genes for MRI. The premise when using reporter genes is that the reporter, that is, the protein giving the signal, is only active when the cell is viable. If the cells proliferate, gene expression is related to the number of cells and thus the signal increases. If the cells die, gene expression stops and the signal is lost. Bioluminescence imaging using luciferases is a good example of how well such systems work [5,6], but the use of optical methods to track cells is not possible in large animals due to limited penetration depth. For MRI, proteins related to iron regulation have been proposed as reporters, where control of iron accumulation in cells could work as a means to generate contrast [7]. Because iron is highly paramagnetic, a greater accumulation in the cells to be tracked could allow them to generate contrast and be distinguished from host cells via magnetic resonance (MR).

Transferrin receptor 1 (TfR1) and ferritins are well known regulators of cellular iron. TfR1 is a transmembrane glycoprotein responsible for internalising iron bound transferrin, which is then released into the cytoplasm and stored in a non-toxic form inside metalloprotein complexes called ferritins [8]. Ferritins store around 20%–30% of the total iron present in an organism and consist of protein complexes formed of two different subunits: a heavy subunit (H) and a light subunit (L). At the gene level, H and L subunits derive from two different genes, ferritin heavy chain 1 (*Fth1*) and ferritin light chain 1 (*Ftl1*), and are located on different chromosomes [9]. Studies have demonstrated that the heavy subunit is the main controller of ferritin function and has a ferroxidase activity centre, which is essential for iron oxidation and incorporation [10,11]. In contrast, the light subunit facilitates the heavy subunit activity and is responsible for iron nucleation and mineralisation. Therefore, the heavy subunit incorporates iron several fold faster than the light subunit [12,13].

These two genes (*TfR1* and *Fth1*) have been previously explored as reporter genes for the MRI of cancer cells [14,15,16,17] and stem cells [18,19,20,21]. In these studies, iron accumulation in the target cells is usually achieved by overexpression of these proteins via chromosomal insertion of the respective genes under the control of a constitutive promoter. Although some of these studies have displayed limited success in animal models, no reports exist so far on the potential of using a combination of these two genes in mesenchymal stem cells (MSCs). More importantly, it is not clear what impact overexpressing these genes has on the cell’s endogenous iron regulation. Additionally, whether the MR contrast that can be achieved with the reporters is any better than what can be obtained by simply supplementing the cell culture medium with high concentrations of iron has not yet been described. Here, we explore the interplay between exogenous and endogenous iron regulatory proteins, and determine the extent to which MRI reporter genes can improve contrast in MSCs.

## 2. Results

**Figure 1:**
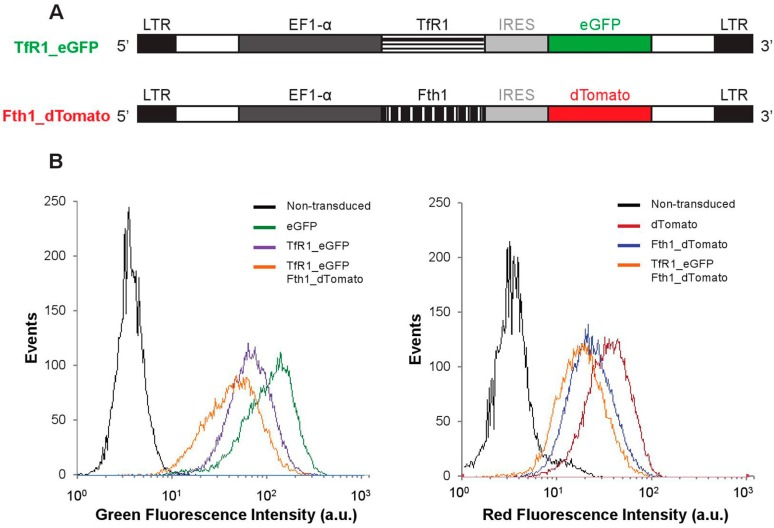
Reporter gene constructs and genomic integration in MSCs. (**A**) Schematic representation of each of the constructs. LTR: long terminal repeat; EF1-α: Elongation factor 1 alpha; (**B**) Fluorescence histograms obtained via flow cytometry showing successful integration of the transgenes.

Two lentiviral plasmid constructs were generated in this work (Figure 1A). In addition to the empty vectors (encoding for *dTomato* and enhanced green fluorescence protein, *eGFP*, but no magnetic reporter gene), constructs encoding bicistronic *TfR1* and *eGFP* mRNAs or *Fth1* and *dTomato* mRNAs were prepared. After transduction with lentiviral particles encoding for each of these constructs (or a combination of *TfR1* and *Fth1*), 95% of the cell population expressed the fluorescent proteins, confirming successful integration of the reporter genes (Figure 1B and Figure S1).

**Figure 2:**
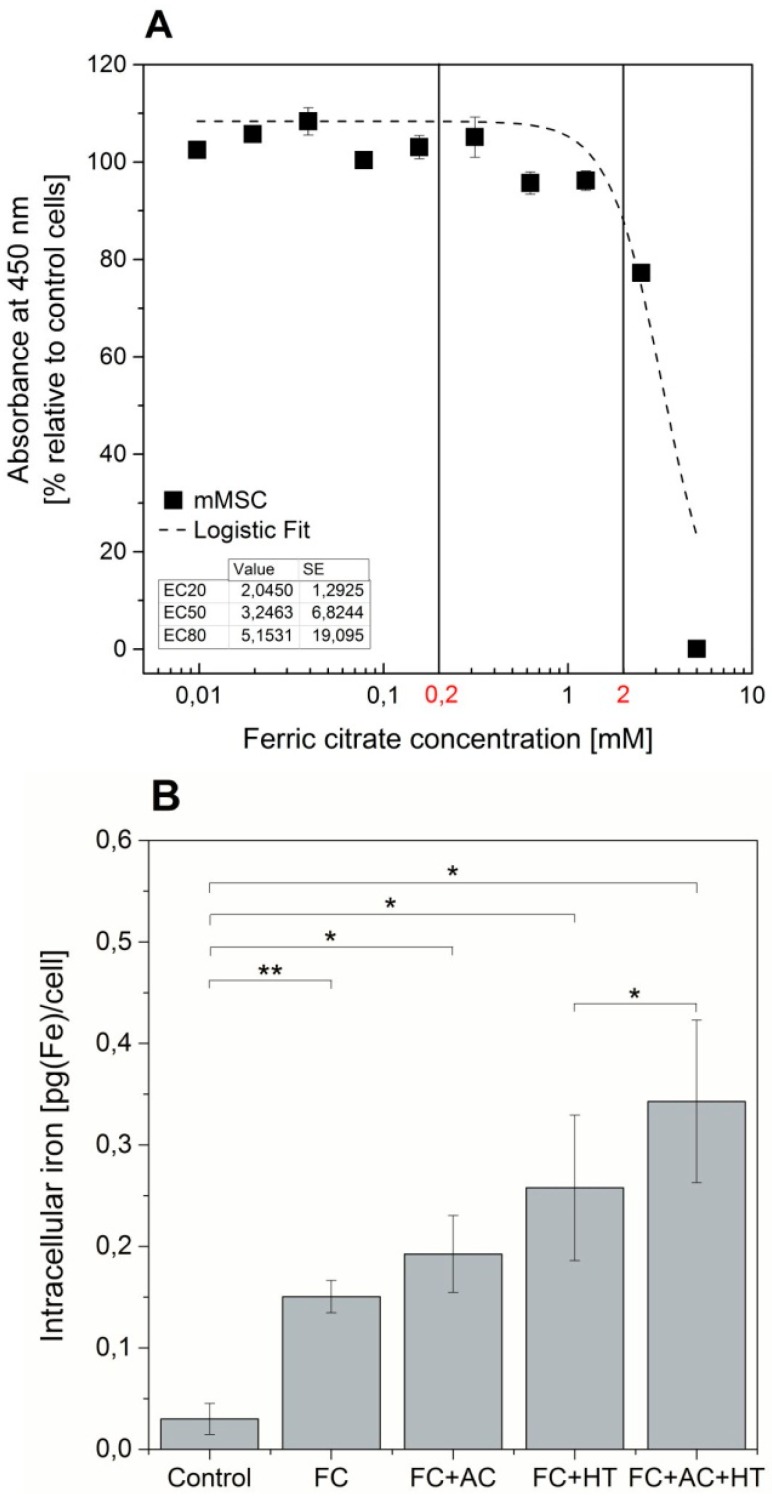
Iron uptake and cytotoxicity. (**A**) Cell viability of MSCs when exposed to different doses of ferric citrate; (**B**) Effect of culture medium supplements on intracellular iron content. FC: 0.2 mM ferric citrate; AC: 50 µM l-ascorbic acid; HT: 1.28 mM human holo-transferrin. Error bars represent SEM (*n =* 3). * denotes *p* < 0.05 and ** *p* < 0.01.

In order to establish the limits of iron supplementation that the MSCs can tolerate, the viability of the cells was evaluated after 24 h exposure to a range of iron concentrations. From the data displayed in Figure 2A, it was found that the inhibitory concentrations (IC) 20, IC50 and IC80 of ferric citrate for this cell line were 2.05, 3.25 and 5.15 mM respectively. Based on these data, a safe labelling concentration of 0.2 mM was chosen. The actual iron internalised by cells when supplemented for 24 h with ferric citrate is displayed in Figure 2B; by simply supplementing the culture medium with ferric citrate the intracellular iron content can be increased from 0.03 to 0.15 pg/cell. We have also investigated the effects of co-supplementing the medium with ascorbic acid, holo-transferrin or a combination of those, each of which allowed a stepwise increase in the intracellular iron content of MSCs (0.19, 0.26 and 0.34 pg/cell respectively).

**Figure 3:**
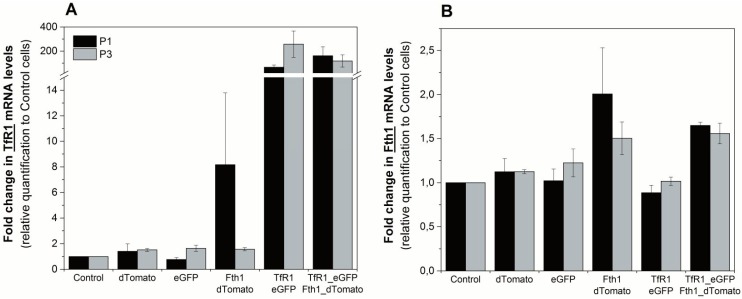
Relative quantification of the reporter gene transcripts at passages 1 and 3 after transduction. Fold change in expression of (**A**) *TfR1* or (**B**) *Fth1*. Expression levels are relative to control (non-transduced) cells. Error bars represent SEM (*n =* 3).

To assess the effects of reporter-gene overexpression on the endogenous regulation of *Fth1* and *TfR1*, we quantified the mRNA transcripts of these genes via RT-qPCR. The relative expression of *TfR1* was over 65-fold higher in cells transduced with the TfR1_eGFP plasmid compared to controls, showing that the reporter gene integration and overexpression was successful (Figure 3A). We also observed an increase in *TfR1* expression for cells that were transduced with Fth1_dTomato only. This suggests that the overexpression of *Fth1* leads to an upregulation of the endogenous transferrin receptor, which is more evident immediately after transduction. Relative changes in the expression of *Fth1* are shown in Figure 3B for the same conditions. Here, we see a small increase in *Fth1* expression for those cells that were transduced with Fth1_dTomato. Cells transduced with TfR1_eGFP alone do not show an increase in *Fth1* levels, suggesting that *TfR1* does not interfere with the transcription of ferritin. Relative expression of *Fth1* in cells transduced with Fth1_dTomato only decreased from 2 to 1.5 with respect to controls from the measurement taken immediately after transduction to that taken at passage 3, which is similar to the trend seen with *TfR1* expression under the same conditions (Figure 3A).

**Figure 4:**
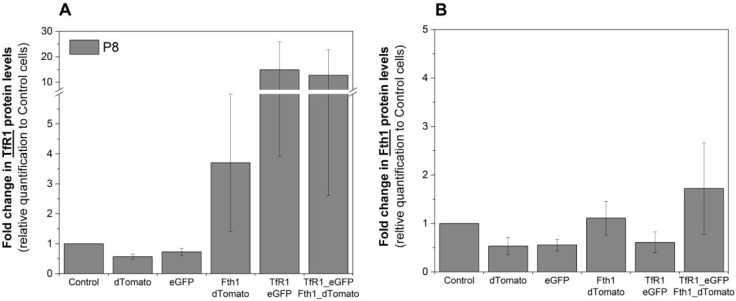
Relative quantification of the reporter proteins. Fold change in the intensity of (**A**) TfR1 or (**B**) Fth1 protein band. Protein levels are relative to the band intensity obtained with control (non-transduced) cells, and total protein quantification was used for data normalisation. Error bars represent SEM (*n =* 3). Western blots are shown in Figure S2.

Western blots were used to assess if the changes in mRNA levels were reflected in protein translation. Relative quantification of TfR1 protein is shown in Figure 4A and is in good agreement with RT-qPCR data, where TfR1 levels are relatively stable for cells transduced with the empty vectors, whereas cells that were transduced with either Fth1_dTomato or TfR1_eGFP displayed increased levels of this protein. Cells transduced with TfR1_eGFP presented a strong increase in TfR1 band intensity (over 12-fold) whereas cells transduced with Fth1_dTomato only displayed a modest increase (3.7-fold) (Figure 4A). The relative increase in Fth1 protein levels (Figure 4B) was small and only seen in cells transduced with Fth1_dTomato, confirming that *TfR1* overexpression does not affect the regulation of this protein.

**Figure 5:**
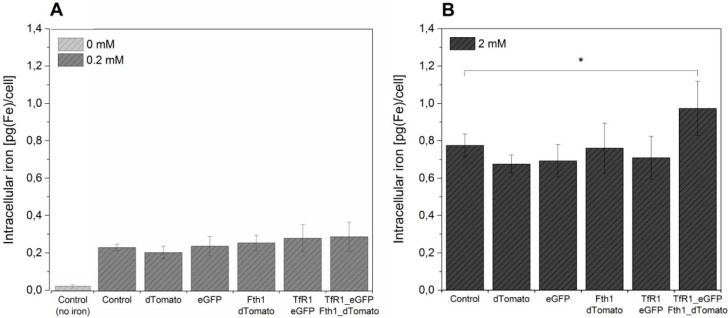
The effect of reporter genes on intracellular iron content of MSCs. Control and transduced cells were supplemented with (**A**) 0.2 mM ferric citrate from 24 h post transduction or (**B**) with 2 mM for 96 h prior to measurement. A control without iron supplementation is also shown. Error bars represent SEM (*n =* 3). Asterisk denotes *p* < 0.05.

The effect of the overexpression of *TfR1* and *Fth1*, both singly and in combination, on intracellular iron content is shown in Figure 5. A significant increase in the amount of iron accumulated by cells is seen by supplementation, but no significant differences were observed between the different conditions, irrespective of the reporter gene system being used. In order to assess if higher quantities of ferric citrate could lead to higher levels of intracellular iron, we have also supplemented cells with 2 mM ferric citrate for 96 h prior to iron measurement. This resulted in an approximately 4-fold increase in intracellular iron for the controls (non-transduced cells and cells transduced with empty vectors) as well as for cells transduced with TfR1_eGFP and Fth1_dTomato alone (~0.2 to ~0.8 pg/cell) without any significant differences between samples. Cells transduced with a combination of both reporter genes displayed a small but significantly higher increase in intracellular iron, reaching 0.95 pg/cell.

**Figure 6:**
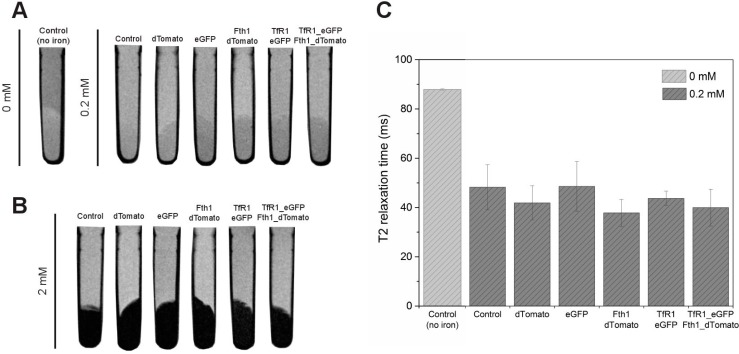
MR contrast of cells expressing *TfR1*, *Fth1* or a combination of these genes. MR images of cells (**A**) non-supplemented (0 mM) and supplemented with 0.2 mM or (**B**) supplemented with 2 mM of ferric citrate. Images obtained with a RARE T_2_-weighted sequence, repetition time = 5000 ms, echo time = 11 ms; (**C**) Relaxation time as measured from a region of interest in the area of the pellets shown in **A**.

The MR contrast of pellets obtained from cells supplemented with each concentration of ferric citrate is shown in Figure 6A. When imaging the pellets with a T_2_-weighted sequence, a noticeable loss of signal (darkening of the cell pellet) is observed for cells supplemented with iron compared to control (non-transduced, not supplemented) cells. The intensity of the signal loss is in good agreement with the intracellular iron content measured; that is, the signal is more hypointense for cells supplemented with 2 mM of ferric citrate. At 0.2 mM of ferric citrate, the hypointense signal is accompanied by a decrease in T_2_ relaxation time (Figure 6B) as measured with T_2_-relaxation maps, with no significant differences between conditions. When cells were supplemented with 2 mM of ferric citrate, the extent of reduction in relaxation time was too great to be quantified.

**Figure 7:**
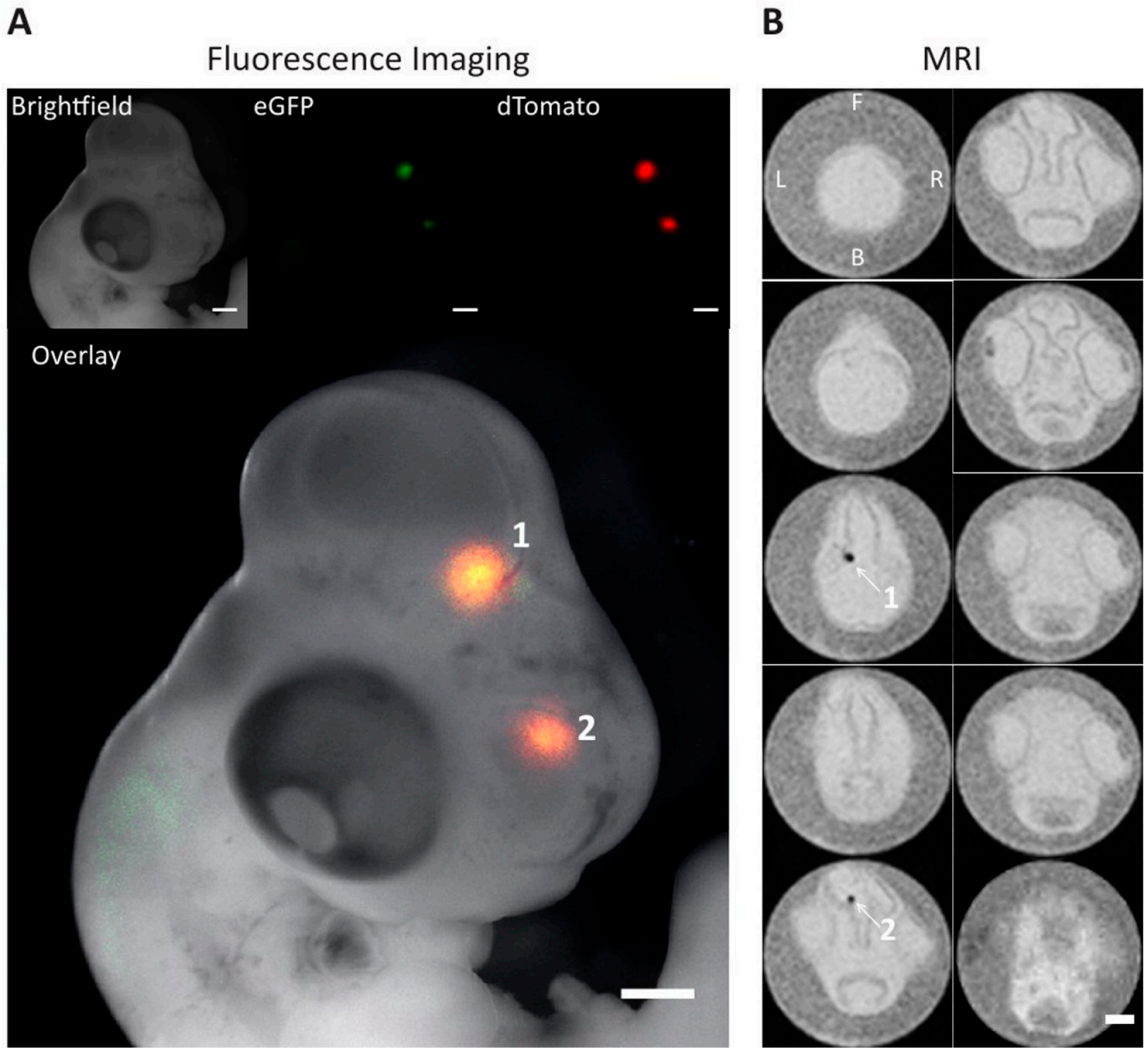
Fluorescence and magnetic resonance (MR) imaging of a chick embryo that received a bolus injection of cells overexpressing *TfR1* and *Fth1*. Cells (2 × 10^5^) were implanted in the midbrain of chick embryos at E3. On E5 chicks were harvested from their eggs, imaged with a fluorescence stereomicroscope and then fixed prior to MR imaging. (**A**) Fluorescence images of the chick embryo and overlay with a brightfield image; (**B**) MR images of transverse sections along the rostrocaudal axis of the embryo; 10 sections are displayed in two columns displaying the head of the embryo. Scale bars correspond to 1 mm. Numbers indicate cell clusters and corresponding T_2_ shortening effect. Position of the embryo: F-front, B-back, L-left, R-right.

Implantation of 2 × 10^5^ cells supplemented with 2 mM of ferric citrate in the midbrain of chick embryos gave rise to clear cell clusters in this organ formed from viable cells as evidenced from fluorescence microscopy images (Figure 7A and Figure S3). The presence of fluorescent proteins 48 h after implantation provides evidence for their survival and integration into the host tissue. The images shown in Figure 7A correspond to a chick embryo that received cells co-transduced with TfR1_eGFP and Fth1_dTomato, where green and red fluorescence are seen from two clusters in different regions of the brain. Each of these spots was easily identified with a Turbo RARE T_2_-weighted sequence as a consequence of their signal loss and corresponding hypointense contrast. Hypointense spots were not observed for cells that were not supplemented with iron (Figure S3E) but were present in all cells that were supplemented with 2 mM ferric citrate, including those cells transduced with empty vectors (Figure S3D,E).

## 3. Discussion

We have generated mesenchymal stem cells expressing *TfR1*, *Fth1* or a combination of these two transgenes with the aim of exploring how each of those iron regulatory genes can impact iron homeostasis and T_2_ contrast in an MSC cell line. The use of these genes has been previously explored in the literature, but the vast majority of the reports involve cancer cell lines [14,15,16,17,21,22,23,24,25] that have a physiology different to stem cells. Furthermore, previous studies have not addressed if overexpression of *TfR1* can have any impact on the expression levels of other iron regulatory proteins.

By using a bicistronic lentiviral vector containing a fluorescence reporter, we were able to confirm successful integration of the transgenes via flow cytometry. In order to bypass the cell’s own post-transcriptional control of the reporter, iron responsive elements (IRE) were removed from both *TfR1* and *Fth1* genes, a method described in one of the earliest reports in the field of magnetic reporter genes [26] and also described by others [18,19,25]. This ensured constitutive expression and translation of the reporter proteins.

In regenerative therapies, the rationale behind the use of reporter genes is often the possibility of identifying the cell’s anatomical location after administration, thus allowing researchers to identify whether the cells have reached (in the case of systemic injection) or remained at (in the case of local injection) their target tissue. When using magnetic resonance (MR), this approach requires high levels of intracellular iron for contrast, which can be facilitated by optimal culture conditions that allow maximal iron uptake. Ferric citrate is a common supplement used to load cells expressing MR-reporters with iron, but concentrations vary in the literature ranging from 0.002 mM [22] up to 2.5 mM [27]. Here, we determined that 0.2 mM is a safe concentration to supplement MSCs that yielded a significant increase in the intracellular iron content. As transferrin is the effective transporter of iron for TfR1 receptor mediated uptake, and because ascorbate has been previously shown to modulate the transferrin uptake pathway [28], we investigated if the addition of these two components to the cell culture medium had an effect in iron uptake. A significant increase in intracellular iron was observed when both were combined with iron citrate, leading to a 1.8-fold increase in intracellular iron when compared with single supplementation with iron citrate alone. Using all three components in combination is therefore an efficient way to increase the cell’s iron levels.

Transduction of MSCs with the *Fth1* gene did not result in substantial increases in the mRNA and protein levels of this gene. Previous research involving the insertion of human ferritin genes into mouse cells [14,15,17,19,22,27] resulted in clear increases in the levels of this gene, but these measured only the exogenous ferritin. Here, we measured total ferritin (endogenous and viral) and although we saw an increase in the expression of this gene right after transduction, the levels were reduced by passage 3, and no differences in protein content were measured by passage 8. Because the endogenous ferritin is controlled by its IRE, it is reasonable to assume that with time, endogenous ferritin expression is downregulated in response to the virally inserted ferritin as a consequence of iron depletion. Interestingly, an increase in *TfR1* expression is observed via RT-qPCR and western blot as a response to the transduction with *Fth1*. This effect has been previously observed for cancer cells lines [14,29] and embryonic stem cells [19] and our results provide evidence that this is also true for adult stem cells. In agreement with the decrease in total ferritin, this increase in *TfR1* is likely to be caused by a reduction in the cell’s labile pool of iron due to sequestration by ferritins, inducing an increase in the expression of the endogenous transferrin receptor, which is expected, given its control by IRE.

The expression of the *TfR1* construct is much more efficient when mRNA and protein levels are considered, with transcript levels stable between different passages and consistent increases in protein levels, the effects being observed up to passage 8 (latest time point studies). Transduction with *TfR1* did not affect *Fth1* expression, suggesting that overexpression of this gene has a smaller impact on iron homeostasis when compared to *Fth1*. The literature on the use of the transferrin receptor as a reporter gene is more limited than that of ferritin, with most of the reports so far focusing on the use of this gene as a membrane receptor to facilitate the uptake of transferrin bound to an iron oxide nanoparticle (IONP) [26,30,31]. Although this is an interesting approach for the targeted delivery of nanoparticles, it does present the same limitations as direct cell labelling of IONPs, such as the transfer of the nanoparticles to host cells upon cell death.

When compared to iron-supplemented controls, cells expressing the reporter genes did not result in an increase in iron accumulation under our supplementation conditions (0.2 mM of ferric citrate combined with ascorbate and holo-transferrin). This was reflected in the T_2_ relaxation time of cell pellets, which were all shorter than non-supplemented controls, but without differences between the use of TfR1_eGFP, Fth1_dTomato or a combination of these. The increase of iron uptake when using genetic reporters is modest when compared to IONPs. For example, when labelling this cell line with iron-oxide based contrast media one can routinely achieve intracellular iron concentrations of over 5 pg[Fe]/cell [32], whereas by using reporter genes we could barely achieve 1 pg[Fe]/cell. Previous research using ferritins suggested that when iron accumulation is considered, the benefits of using reporter genes are very limited. Reported increases in intracellular accumulation of iron as a response to transgenes vary from around 20% [14,25] up to 100% with respect to controls when using engineered mitochondrial ferritins [24]. Although our results suggest an increase of about 20% for cells transduced with a combination of TfR1_eGFP and Fth1_dTomato when cultured with 0.2 mM ferric citrate, this was not significant. Increasing the amount of ferric citrate in the culture medium by 10-fold (2 mM) was shown to be a much more effective way of increasing the cell’s intracellular iron content, resulting in ~4-fold increase in intracellular iron content for all conditions. Here, the combination of TfR1_eGFP and Fth1_dTomato resulted in a significant increase in iron content of about 25%, but this is still modest when compared to the effects of medium supplementation only. These results were reflected in the imaging of cell pellets supplemented with 2 mM ferric citrate, which showed more enhancement of transverse relaxation compared to cells supplemented with 0.2 mM ferric citrate. In fact, relaxation under these conditions was too rapid to be quantified with our scanning protocols.

We confirmed the feasibility of detecting these cells *in vivo* by imaging chick embryo brains which had been injected with 2 × 10^5^ cells. Because of the small size of the embryo, we were able to verify the viability of the cells (via the expression of fluorescence proteins) and their anatomical location with a stereomicroscope prior to MR imaging. In all cases, fluorescence from the constructs was detectable 48 h after injection with cells forming one or more clusters within the brain. We found an excellent correlation between the anatomical location as imaged via fluorescence imaging and the hypointense regions detected via MR, and such clusters were detected irrespective of the construct used.

It is striking that the literature on the use of MR reporter genes for stem cells is so small, given that the imaging and tracking of cells is one of the great challenges in cell-based regenerative medicine therapies. Recent reports using stem cells involved either the imaging of tumours formed by embryonic stem cells [19], very localised labelling of endogenous cells in the brain [21] or the imaging of a bolus injection of myoblasts into the heart [20] or brain [18]. Similarly to what we have done here, all these studies involved the imaging of a concentrated mass of cells localised in a small volume of tissue. The use of reporter genes for stem cell-based therapies that involve a systemic administration of the cells, as opposed to a local administration, however, will probably be much more limited. Because the increase in iron accumulation using reporter genes is so small, it is unlikely that this would prove a sensitive enough approach unless a large number of cells are concentrated in a very small volume. The low intracellular iron levels is compounded by ferritin’s intrinsic low magnetic moment when compared to IONPs [27].

It is also of concern that overexpression of some of these genes might alter the cell’s phenotype possibly leading to adverse consequences when stem cell therapies are considered. When cells were transduced with the *Fth1* reporter, a noticeable change in the morphology and the rate of proliferation of the cells was observed when the culture medium was not supplemented with iron (Figure S4), suggesting a toxic effect related to imbalances in iron homeostasis. It is therefore not clear what the consequences would be if those cells were used for regenerative therapies in an environment depleted of iron, which could be the case when administering cells *in vivo*. Interestingly, a study involving neuroprogenitor cells also reported changes in cell morphology when *Fth1* was used as a reporter gene [18] and changes in phenotype have also been reported for HeLa [33] and erythroleukemia cells [34] overexpressing ferritin heavy chain. Furthermore, a slower growth of nasopharyngeal carcinoma tumours expressing ferritin heavy chain has also been reported [17]. These changes in phenotype are likely to be related to a reduction on the cell’s labile pool of iron as a consequence of iron sequestration by ferritins and must be taken into account, not only when therapeutic cells are considered, but also in cancer research, as it can clearly alter the cell’s behaviour.

In summary, our evidence suggests that MSCs can tolerate overexpression of ferritin heavy chain-1, transferrin receptor-1, or a combination of these genes provided that the culture medium is supplemented with enough iron to prevent phenotypic changes of the cells. Overexpression of transferrin receptor has a lower impact on iron homeostasis than ferritin, which upregulates the expression of endogenous transferrin receptor and is likely to downregulate the expression of endogenous ferritin. The efficacy of these reporter genes in stimulating intracellular iron accumulation *in vitro* is limited, with a small advantage seen when ferritin and transferrin receptor are used in conjunction. This system could be of benefit for the long-term monitoring of large clusters of cells, where the transgenes could possibly lead to increased intracellular iron content in relation to the surrounding tissues as has been explored in previous studies [18,19,20,21]. However, when imaging protocols that involve the detection of cells immediately after implantation are considered, supplementing the culture medium with adequate iron sources has a more significant impact than the use of reporter genes, with a combination of ferric citrate, ascorbate and holo-transferrin providing maximal intracellular iron concentrations.

## 4. Experimental Section

### 4.1. Cell Culture and Iron Supplementation

The multipotent murine MSC D1 cell line (CRL-12424™, ATCC, Teddington, UK) was cultured in Dulbecco’s Modified Eagle’s Medium (DMEM) containing 10% fetal calf serum (FCS) at 37 °C under a humidified atmosphere with 5% CO_2_. All culture media and supplements were purchased from Sigma-Aldrich, Gillingham, UK, unless stated otherwise. For measurement of viability, cells (5 × 10^3^ cells/well in a 96-well plate) were supplemented with a range of concentrations of ferric citrate (9.8–5000 μM) for 24 h (*n =* 3). Cell viability was assessed with a WST-8 compound, according to manufacturer’s instructions (Sigma, Cell Counting Kit-8). For iron supplementation, cells (5 × 10^4^ cells/well in a 6-well plate) were exposed to different combinations of ferric citrate (0.2 mM), l-ascorbic acid (50 µM) and human holo-transferrin (1.28 mM), for 4 days. Intracellular iron content was measured using a ferrozine-based assay (described below).

### 4.2. Generation of Lentiviral Constructs and Viral Production

Mouse *TfR1* (NM_011638.4) and *Fth1* (NM_010239.2) cDNAs were synthesised from a RNA sample of the MSC D1 line. Iron responsive elements were identified using SIREs Web Server 2.0 software [35] and excluded from amplification, to avoid the cell’s post-transcriptional control of these genes. *TfR1* and *Fth1* cDNAs were then cloned into pHIV-eGFP (Addgene, Cambridge, MA, USA, 21373) and pHIV-dTomato (Addgene, 21374), respectively. In both lentiviral vectors, the elongation factor-1 alpha (EF1-α) was used as a constitutive promoter and the expression of the fluorescent protein was mediated via an internal ribosome entry site (IRES). For viral production, individual lentiviral vectors (transfer plasmids) were transiently co-transfected with the packaging plasmid psPAX2 (Addgene, 12260) and the envelope plasmid pMD2.G (Addgene, 12259), at mass ratio 4:2:1, into a producer cell line (human embryonic kidney 293TN). Transfection methods and titration of lentiviral particles were performed as previously described [36].

### 4.3. Transduction and Evaluation of Reporter Gene Expression

#### 4.3.1. Transduction with Lentiviral Particles

Cells (10^3^ cells/well in a 48-well plate) were transduced at multiplicity of infection (MOI) of 50 by incubation with lentiviral particles and polybrene (8 μg/mL) for 16 h. Transduction of cells was performed in three independent experiments (*n =* 3). Iron supplements were added 24 h post transduction (0.2 mM ferric citrate, 50 µM l-ascorbic acid and 1.28 mM human holo-transferrin) and for all quantitative measurements non-transduced cells were harvested at an equivalent passage as controls. After transduction, cells were expanded for 7 days and then subcultured every 3–5 days. Cells were not used beyond passage 8 after transduction. Expression of *eGFP* and *dTomato* was assessed with a BD FACScalibur instrument (BD Biosciences, Oxford, UK) using a 488 nm excitation laser and appropriate detectors according to transgene.

#### 4.3.2. RT-qPCR and Western Blot

For RT-qPCR, 9 × 10^4^ cells were collected at passages 1 and 3 after transduction. The cDNA synthesis and RT-qPCR (CFX Connect™ Real-Time PCR Detection System, Bio-Rad, Hemel Hempstead, UK) were performed according to manufacturer’s instructions (Fast SYBR^®^ Green Cells-to-CT™ Kit, Life Technologies, Paisley, UK). Mouse *Tbp* and *Pgk1* were used as reference genes for data normalisation and primers for all transcripts are shown in the supplementary material. Data were analysed using CFX System Test Software (Bio-Rad).

For Western blotting, 10^6^ cells were collected at passage 8 after transduction. Proteins were extracted, quantified, run on a gel using a Novex^®^ NuPAGE^®^ SDS-PAGE Gel System (Life Technologies) and wet transferred to Immobilon^®^ FL Transfer Membranes (PVDF, Millipore, Watford, UK). Anti-ferritin heavy chain 1 (Abcam, Cambridge, UK, ab65080), anti-transferrin receptor 1 (Abcam, ab84036) and anti-actin (Abcam, ab1801) antibodies were used as primary antibodies and IRDye 680RD Donkey anti-Rabbit (Licor, Cambridge, UK, 926-68073) as a secondary antibody. Total protein gels were used for data normalisation and actin was used as a reference to confirm data normalisation. More information about the Western blot protocols are provided in the supplementary material.

#### 4.3.3. Iron Uptake and Quantification

Cells (5 × 10^4^ cells/well in a 6-well plate) were incubated with 50 µM l-ascorbic acid, 1.28 mM human holo-transferrin and either 0.2 or 2 mM of ferric citrate and then processed for iron quantification. 10^6^ cells were digested with 1:1 ratio of 1.2 M HCl and 4.5% KMnO_4_, for 2 h, 60 °C. Then, 20 μL of ferrozine reagent (5 M ammonium acetate, 2 M ascorbic acid, 6.5 mM 3-(2-Pyridyl)-5,6-diphenyl-1,2,4-triazine-*p*,*p*′-disulfonic acid monosodium salt hydrate and 15.4 mM neocuproine) were added and the reaction was left to develop for 30 min. Absorbance was read at 570 nm and iron was quantified based on a standard curve prepared with an iron standard (TraceCERT™, Sigma).

### 4.4. Fluorescence and Magnetic Resonance (MR) Imaging

#### 4.4.1. Cell Pellet Phantoms

A total of 10^7^ cells were incubated under the same conditions used for iron quantification, fixed with 4% formaldehyde and transferred to 0.2 mL polypropylene tubes. After a 30 min centrifugation at 13,400× *g*, 1% low-melting agarose was placed on top of the cell pellet.

#### 4.4.2. Preparation of Chick Embryos

MSCs (2 × 10^5^) were incubated with l-ascorbic acid, human holo-transferrin and 2 mM ferric citrate for 3 days, harvested and suspended in saline containing 15% deoxyribonuclease I and 15% fast green. The cells were then injected into the ventricle cavity of White Leghorn chicken eggs, at embryonic day 3 (E3), using a microcapillary pipette. Eggs were kept at 37 °C and 40% humidity. All work followed standard ethical guidelines according to UK regulations (Consolidated version of ASPA 1986). At E5, embryos were harvested and bright-field and fluorescence images were acquired using a Leica M165FC stereomicroscope. Embryos were then fixed with 4% formaldehyde, washed, embedded in 1% low-melting agarose and mounted in 0.5 mL microcentrifuge tubes.

#### 4.4.3. MR Imaging

A 7 T Avance III MRI instrument (Bruker, Coventry, UK) was used to image cell phantoms and chick embryos with a 38 mm transmit/receive quadrature volume coil. To obtain the transversal relaxation time of cell pellets, maps were generated using a modified Rapid Acquisition with Refocused Echoes (RARE) sequence with repetition times (TR): 5000, 3000, 1500, 800, 400 and 200 ms, and echo times (TE): 11, 22, 55, 77 and 99 ms. Anatomical images were obtained with a high resolution TurboRARE T_2_-weighted sequence with the following parameters: field of view 30 × 30 mm, matrix 256 × 256, slice thickness 0.5 mm, effective TE 33 ms, RARE factor 8, TR 2741.9 ms, averages 10, flip angle 135°.

## Supplementary Materials

Supplementary materials can be found at http://www.mdpi.com/1422-0067/16/07/15481/s1.

## References

[1] Rodriguez-Porcel M., Wu J.C., Gambhir S.S., Girard L. (2009). Molecular imaging of stem cells. Stembook.

[2] Naumova A.V., Modo M., Moore A., Murry C.E., Frank J.A. (2014). Clinical imaging in regenerative medicine. Nat. Biotechnol..

[3] Taylor A., Wilson K.M., Murray P., Fernig D.G., Levy R. (2012). Long-term tracking of cells using inorganic nanoparticles as contrast agents: Are we there yet?. Chem. Soc. Rev..

[4] Bulte J.W.M. (2009). *In vivo* MRI cell tracking: Clinical studies. Am. J. Roentgenol..

[5] De Almeida P.E., van Rappard J.R.M., Wu J.C. (2011). *In vivo* bioluminescence for tracking cell fate and function. Am. J. Physiol. Heart Circ. Physiol..

[6] Kim J., Kalimuthu S., Ahn B.-C. (2015). *In vivo* cell tracking with bioluminescence imaging. Nucl. Med. Mol. Imaging.

[7] Vandsburger M.H., Radoul M., Cohen B., Neeman M. (2013). MRI reporter genes: Application to imaging of cell survival, proliferation, migration, and differentiation. NMR Biomed..

[8] Muckenthaler M.U., Galy B., Hentze M.W. (2008). Systemic iron homeostasis and the iron-responsive element/iron-regulatory protein (IRE/IRP) regulatory network. Ann. Rev. Nutr..

[9] Arosio P., Ingrassia R., Cavadini P. (2009). Ferritins: A family of molecules for iron storage, antioxidation and more. Biochim. Biophys. Acta.

[10] Chasteen N.D., Harrison P.M. (1999). Mineralization in ferritin: An efficient means of iron storage. J. Struct. Biol..

[11] Levi S., Luzzago A., Cesareni G., Cozzi A., Franceschinelli F., Albertini A., Arosio P. (1988). Mechanism of ferritin iron uptake: Activity of the H-chain and deletion mapping of the ferro-oxidase site. A study of iron uptake and ferro-oxidase activity of human liver, recombinant H-chain ferritins, and of two H-chain deletion mutants. J. Biol. Chem..

[12] Koorts A.M., Viljoen M. (2007). Ferritin and ferritin isoforms I: Structure-function relationships, synthesis, degradation and secretion. Arch. Physiol. Biochem..

[13] Santambrogio P., Levi S., Cozzi A., Rovida E., Albertini A., Arosio P. (1993). Production and characterization of recombinant heteropolymers of human ferritin H and L chains. J. Biol. Chem..

[14] Aung W., Hasegawa S., Koshikawa-Yano M., Obata T., Ikehira H., Furukawa T., Aoki I., Saga T. (2009). Visualization of *in vivo* electroporation-mediated transgene expression in experimental tumors by optical and magnetic resonance imaging. Gene Ther..

[15] Kim H.S., Cho H.R., Choi S.H., Woo J.S., Moon W.K. (2010). *In vivo* imaging of tumor transduced with bimodal lentiviral vector encoding human ferritin and green fluorescent protein on a 1.5 t clinical magnetic resonance scanner. Cancer Res..

[16] Choi S.H., Cho H.R., Kim H.S., Kim Y.H., Kang K.W., Kim H., Moon W.K. (2012). Imaging and quantification of metastatic melanoma cells in lymph nodes with a ferritin mr reporter in living mice. NMR Biomed..

[17] Feng Y., Liu Q., Zhu J., Xie F., Li L. (2012). Efficiency of ferritin as an MRI reporter gene in NPC cells is enhanced by iron supplementation. J. Biomed. Biotechnol..

[18] Deans A., Wadghiri Y., Bernas L., Yu X., Rutt B., Turnbull D.H. (2006). Cellular MRI contrast via coexpression of transferrin receptor and ferritin. Magn. Reson. Med..

[19] Liu J., Cheng E., Long R., Wang Y., Cheng P., Yang J., Wu D., Mao H., Chan A. (2009). Noninvasive monitoring of embryonic stem cells *in vivo* with MRI transgene reporter. Tissue Eng. C Methods.

[20] Naumova A.V., Reinecke H., Yarnykh V., Deem J., Yuan C., Murry C.E. (2010). Ferritin overexpression for noninvasive magnetic resonance imaging-based tracking of stem cells transplanted into the heart. Mol. Imaging.

[21] Vande Velde G., Raman Rangarajan J., Vreys R., Guglielmetti C., Dresselaers T., Verhoye M., Van der Linden A., Debyser Z., Baekelandt V., Maes F. (2012). Quantitative evaluation of MRI-based tracking of ferritin-labeled endogenous neural stem cell progeny in rodent brain. Neuroimage.

[22] Genove G., DeMarco U., Xu H., Goins W., Ahrens E. (2005). A new transgene reporter for *in vivo* magnetic resonance imaging. Nat. Med..

[23] Iordanova B., Goins W.F., Clawson D.S., Hitchens T.K., Ahrens E.T. (2013). Quantification of HSV-1-mediated expression of the ferritin MRI reporter in the mouse brain. Gene Ther..

[24] Iordanova B., Hitchens T.K., Robison C.S., Ahrens E.T. (2013). Engineered mitochondrial ferritin as a magnetic resonance imaging reporter in mouse olfactory epithelium. PLoS ONE.

[25] Iordanova B., Robison C.S., Ahrens E.T. (2010). Design and characterization of a chimeric ferritin with enhanced iron loading and transverse NMR relaxation rate. J. Biol. Inorg. Chem..

[26] Weissleder R., Moore A., Mahmood U., Bhorade R., Benveniste H., Chiocca E.A., Basilion J.P. (2000). *In vivo* magnetic resonance imaging of transgene expression. Nat. Med..

[27] Velde G.V., Rangarajan J.R., Toelen J., Dresselaers T., Ibrahimi A., Krylychkina O., Vreys R., van der Lindenv A., Maes F., Debyser Z. (2011). Evaluation of the specificity and sensitivity of ferritin as an MRI reporter gene in the mouse brain using lentiviral and adeno-associated viral vectors. Gene Ther..

[28] Lane D.J.R., Chikhani S., Richardson V., Richardson D.R. (2013). Transferrin iron uptake is stimulated by ascorbate via an intracellular reductive mechanism. Biochim. Biophys. Acta.

[29] Iordanova B., Ahrens E.T. (2012). *In vivo* magnetic resonance imaging of ferritin-based reporter visualizes native neuroblast migration. Neuroimage.

[30] Wang K., Wang K., Shen B., Huang T., Sun X., Li W., Jin G., Li L., Bu L., Li R. (2010). MR reporter gene imaging of endostatin expression and therapy. Mol. Imaging Biol..

[31] Ichikawa T., Högemann D., Saeki Y., Tyminski E., Terada K., Weissleder R., Chiocca E.A., Basilion J.P. (2002). MRI of transgene expression: Correlation to therapeutic gene expression. Neoplasia.

[32] Taylor A., Herrmann A., Moss D., Sée V., Davies K., Williams S., Murray P. (2014). Assessing the efficacy of nano- and micro-sized magnetic particles as contrast agents for MRI cell tracking. PLoS ONE.

[33] Cozzi A., Corsi B., Levi S., Santambrogio P., Albertini A., Arosio P. (2000). Overexpression of wild type and mutated human ferritin H-chain in hela cells: *In vivo* role of ferritin ferroxidase activity. J. Biol. Chem..

[34] Picard V., Renaudie F., Porcher C., Hentze M.G.B., Beaumont C. (1996). Overexpression of the ferritin H subunit in cultured erythroid cells changes the intracellular iron distribution. Blood.

[35] Campillos M., Cases I., Hentze M., Sanchez M. (2010). Sires: Searching for iron-responsive elements. Nucleic Acids Res..

[36] Kutner R., Zhang X., Reiser J. (2009). Production, concentration and titration of pseudotyped HIV-1-based lentiviral vectors. Nat. Protoc..

