# Supplementary material for: Overexpression of the MRI Reporter Genes Ferritin and Transferrin Receptor Affect Iron Homeostasis and Produce Limited Contrast in Mesenchymal Stem Cells
Source: Int J Mol Sci. 2015 Jul 8;16(7):15481–96. doi: 10.3390/ijms160715481 (PMC4519909; doi:10.3390/ijms160715481)
Supplement: Supplementary file 1 [file ijms-16-15481-s001.pdf]

Supplementary Information

Primer Pairs Used for RT-qPCR

Primer	Forward Primer (5′–3′)	Reverse Primer (5′–3′)	Product (bp)
TATA box Binding Protein (*Tbp*)	AATAGTGATGCTGGGCACTGCGG	GTGCGTCAGGCGTTCGGTGG	113
Phosphoglycerate kinase 1 (*Pgk1*)	CTGACTTTGGACAAGCTGGACG	GCAGCCTTGATCCTTTGGTTG	110
Ferritin heavy chain-1 (*Fth1*)	TGAGGAGAGGGAGCATGCCGA	CCAGTCATCACGGTCTGGTTT	100
Transferrin Receptor-1 (*Tfr1*)	TGAGTGGCTACCTGGGCTAT	CTCCTCCGTTTCAGCCAGTT	74
Construct specific ferritin heavy chain-1_IRES (*Fth1_IRES*)	TACGCAAGATGGGTGCCCCTGA	ATTCCAAGCGGCTTCGGCCA	140
Construct specific transferrin receptor-1_IRES (*TfR1_IRES*)	GGGAGTCGCAAATGCCCTCT	ATTCCAAGCGGCTTCGGCCA	113

Western Blot Methods

Proteins were extracted with a lysis buffer on ice with rotation for 30 min; centrifuged for 10 min at 13,400× *g* and the protein in the supernatant was quantified following instructions of Pierce™ BCA Protein Assay Kit (Thermo Scientific, Warrington, UK). Then, 10 µg of protein were mixed with 1× NuPAGE LDS Sample buffer (Life Technologies), 1× NuPAGE Reducing Agent (Life Technologies, Paisley, UK) and water up to a volume of 25 µL and subsequently heated at 70 °C for 10 min. Proteins (5 µg) were run in two NuPAGE® Novex® 4%–12% Bis-Tris Protein Gel (Life Technologies), where one was used for antibody incubation and another one for total protein quantification. Odyssey Protein Molecular Weight Marker (Licor, Cambridge, UK) was included in both gels and protein electrophoresis was performed in 1× MOPS running buffer (Life Technologies), 200 V constant, 50 min. For one gel, wet transfer was performed onto Immobilon®-FL Transfer Membranes (PVDF, Millipore, Watford, UK), pre-activated for 30 s in 100% methanol, and proteins were transferred at 30 V constant, 1 h. The membrane was then blocked with Odyssey® Blocking Buffer (Licor), 1 h, and incubated with the following antibodies: anti-ferritin heavy chain-1 (Abcam, Cambridge, UK, ab65080; 1:500 dilution), anti-transferrin receptor-1 (Abcam, ab84036; 1:1000 dilution) and anti-actin (Abcam, ab1801; 1:1500 dilution), diluted in the same blocking buffer, overnight, at 4 °C. Then, the membrane was washed with 1× PBS and incubated with secondary antibody IRDye 680RD Donkey anti-Rabbit IgG (H + L) (Licor, 926-68073; 1:15,000 dilution) for 1 h, at RT. For total protein quantification, the remaining gel was incubated with InstantBlue™ (Expedeon, Swavesey, UK) for 1 h, washed overnight with water and imaged under the same conditions. Total protein gels were used for data normalisation when quantifying proteins. Actin was used as a reference protein to confirm data normalisation. The membranes and stained gels were imaged in LICOR Odyssey® Sa Infrared imaging System (Licor), 700 nm channel, 200 µm resolution and intensity of 5.0. Images were processed and analysed using Image Studio™ Lite (Licor), Version 3.1.

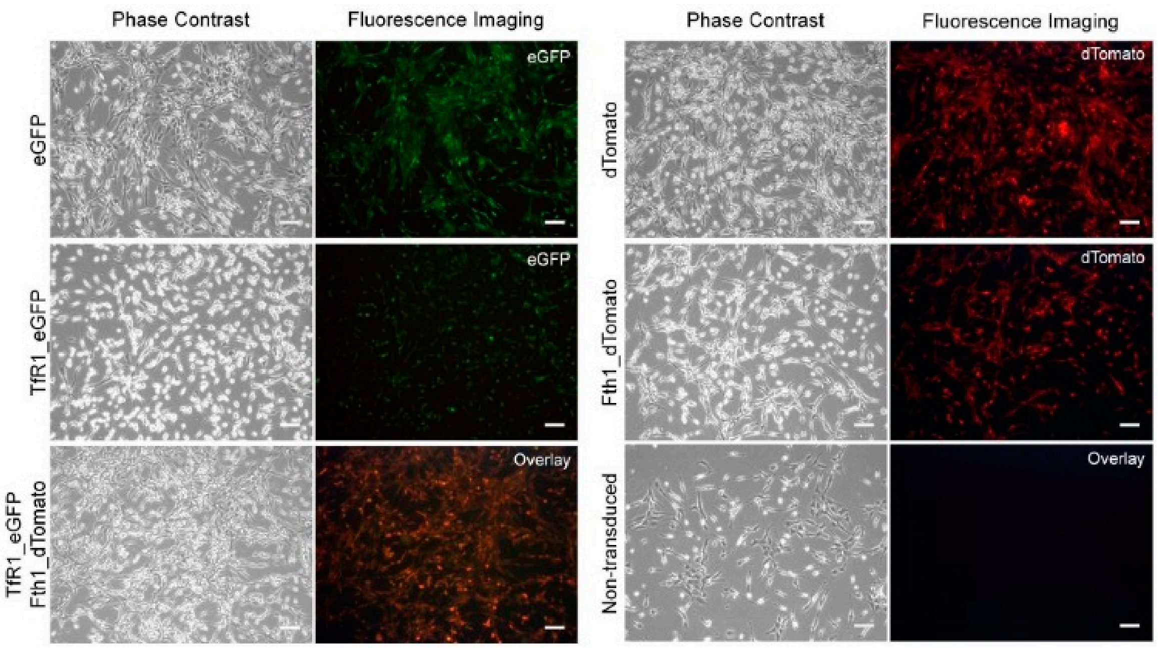

Figure S1. Brightfield and fluorescence Images of MSC D1 cells transduced with empty vectors (*eGFP* or *dTomato*) or vectors containing the reporter genes (TfR1_eGFP and/or Fth1_dTomato). All images were acquired with the same exposure conditions. For double transduced cells an overlay of green and red fluorescence channels is shown. Scale bars correspond to 100 µm.

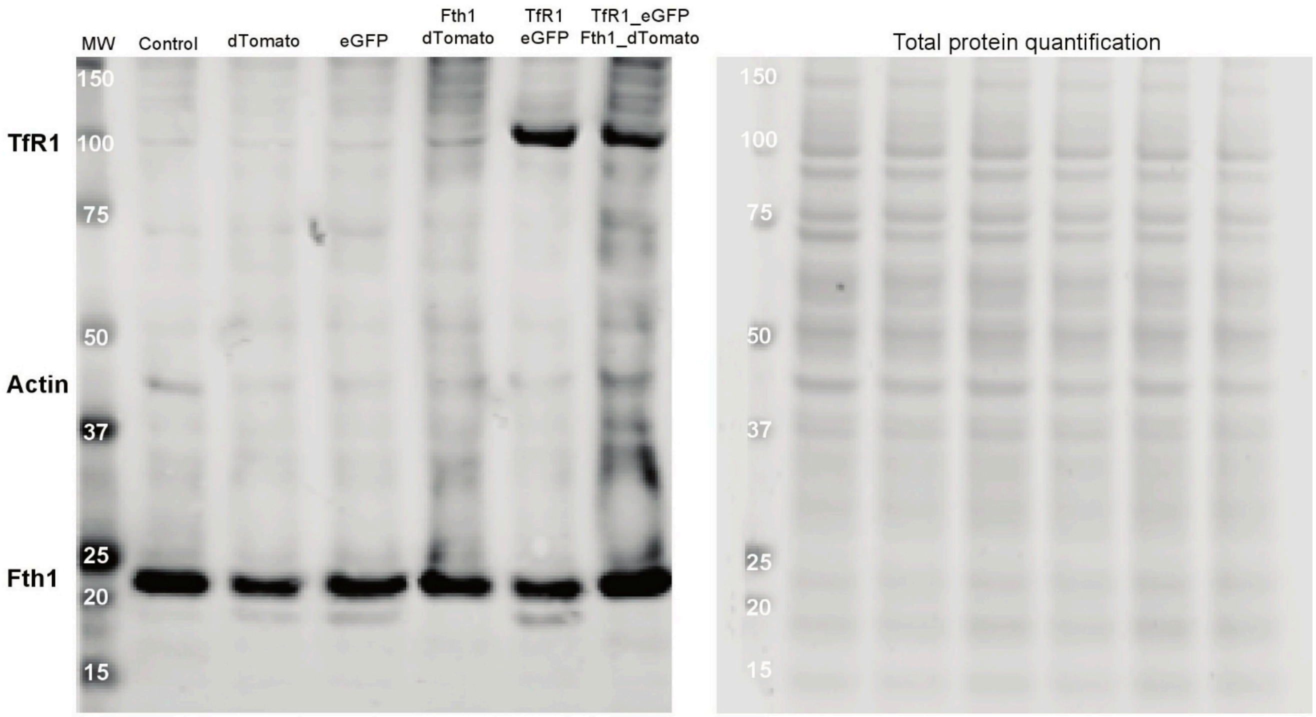

Figure S2. Representative western blot gels. Data was normalised using total protein content and confirmed using the actin band intensity.

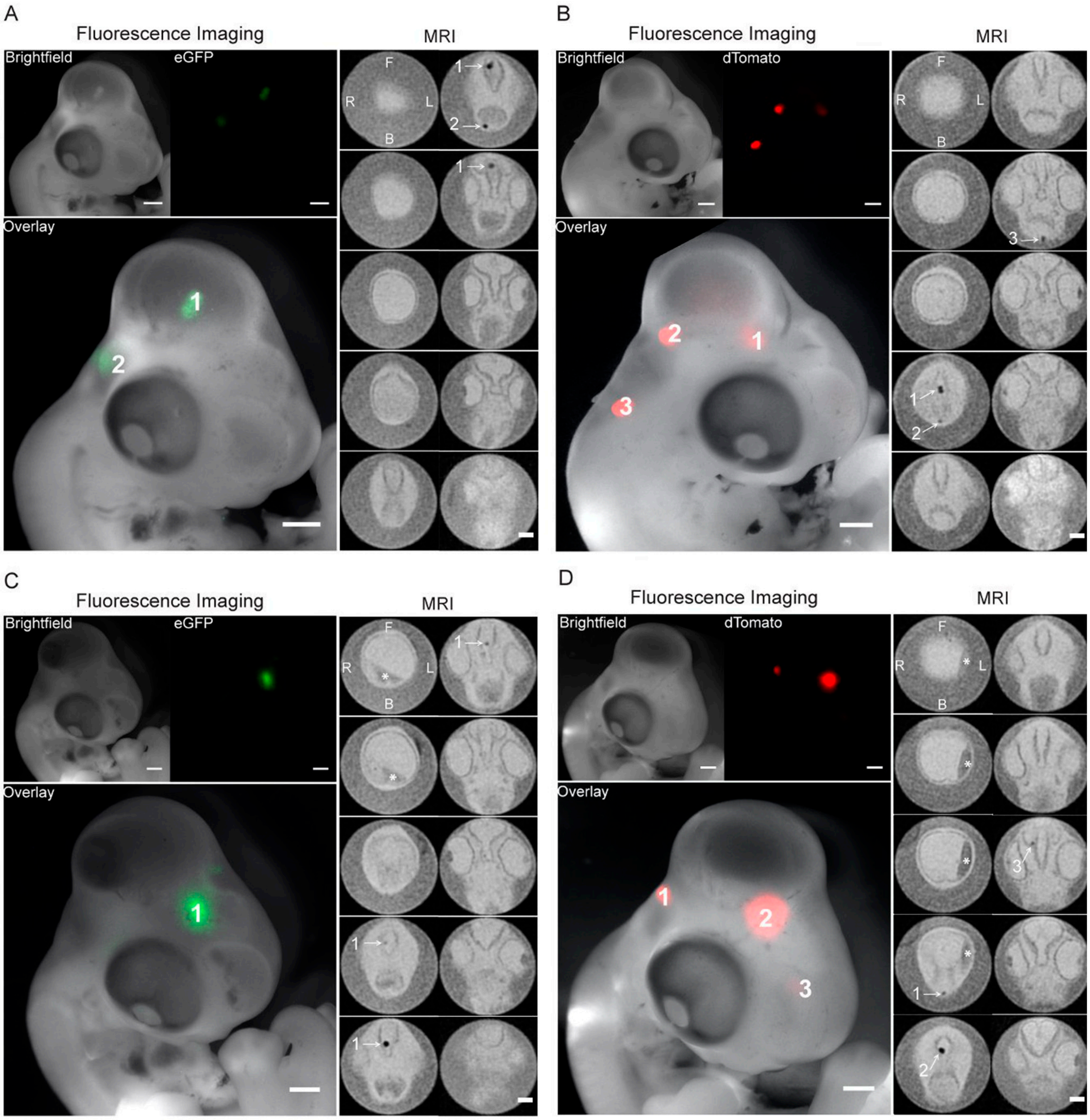

Figure S3. *Cont.*

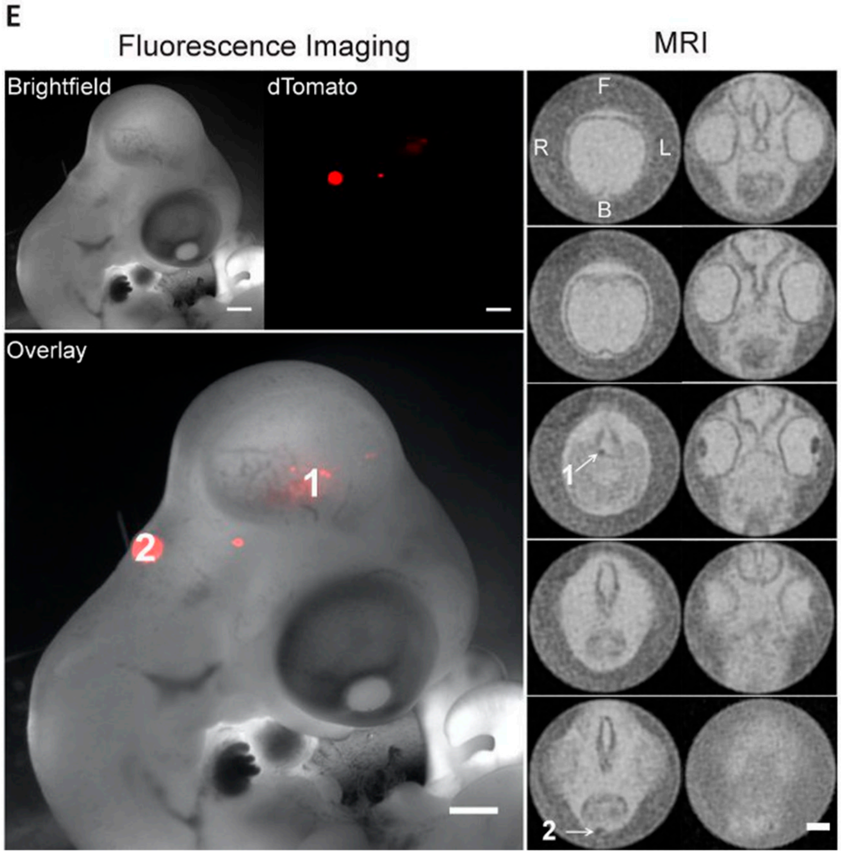

Figure S3. Fluorescence and MR imaging of chick embryos that received a bolus injection of cells overexpressing *TfR1* (**A**) or *Fth1* (**B**) or transduced only with empty vectors, containing either *eGFP* (**C**) or *dTomato* (**D**,**E**). All conditions were incubated with iron supplements (including 2 mM ferric citrate) for 3 days prior to injection, with the exception of E, where no supplementation was added to the culture medium. 2×10^5-cells were implanted in the midbrain of chick embryos at E3. On E5 chicks were harvested from their eggs, imaged with a fluorescence stereoscope and then fixed prior to MR imaging. Fluorescence images and overlay with brightfield are presented on left panel for each condition. MR images correspond to transverse sections along the rostrocaudal axis of the embryo; 10 sections are displayed in two columns displaying the head of the embryo. Scale bars correspond to 1 mm. Numbers indicate cell clusters and corresponding T_2 shortening effect. Position of the embryo: F-front, B-back, L-left, R-right. * corresponds to blood spots visible through brightfield imaging.

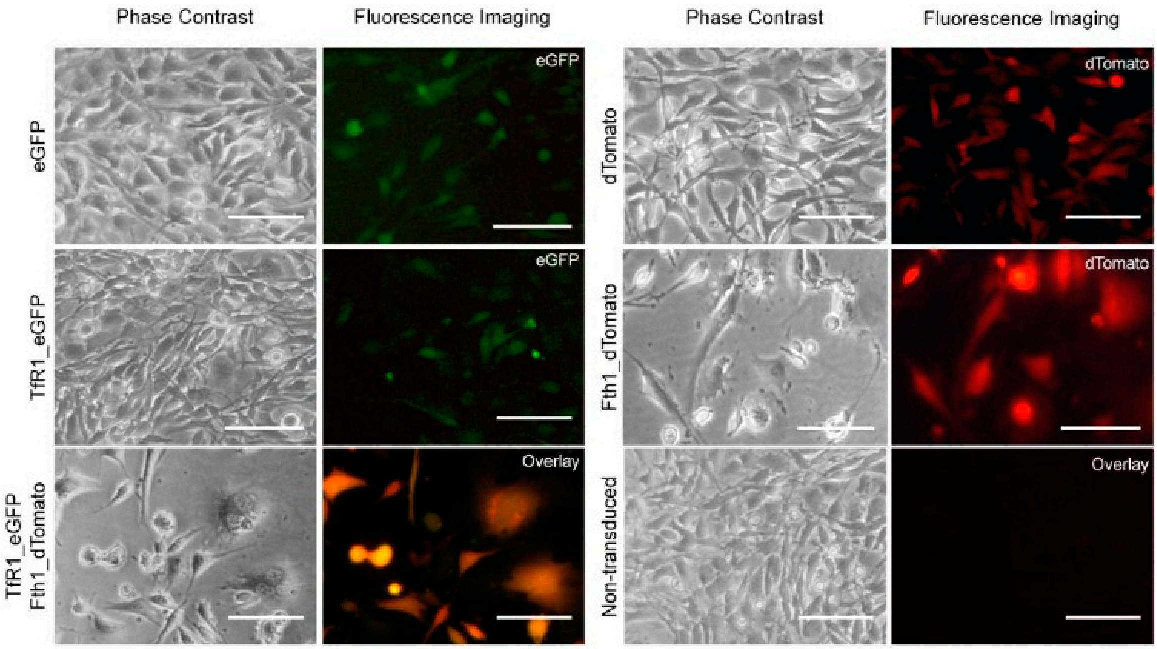

Figure S4. Bright-field and fluorescence Images of MSC D1 cells transduced with empty vectors (*eGFP* or *dTomato*) or vectors containing the reporter genes (TfR1_eGFP and/or Fth1_dTomato) as acquired 7 days after transduction and in the absence of iron supplementation. All images were acquired with the same exposure conditions. Cells transduced with the *Fth1* transgene (Fth1_dTomato and TfR1_eGFP + Fth1_dTomato) proliferate slower and presented morphological changes in the absence of iron supplementation For double transduced cells an overlay of green and red fluorescence channels is shown. Scale bars correspond to 100 μm.
